# Mercury exposure, nutritional deficiencies and metabolic disruptions may affect learning in children

**DOI:** 10.1186/1744-9081-5-44

**Published:** 2009-10-27

**Authors:** Renee Dufault, Roseanne Schnoll, Walter J Lukiw, Blaise LeBlanc, Charles Cornett, Lyn Patrick, David Wallinga, Steven G Gilbert, Raquel Crider

**Affiliations:** 1 United Tribes Technical College, Bismarck, ND, USA; 2 Department of Health and Nutrition Sciences, Brooklyn College of CUNY, Brooklyn, NY, USA; 3 Departments of Neuroscience and Ophthalmology, LSU Neuroscience Center. Louisiana State University Health Sciences Center, New Orleans, LA, USA; 4 Carl Hayden Bee Research Center, Tucson, AZ, USA; 5 Department of Chemistry and Engineering Physics, University of Wisconsin-Platteville, Platteville, WI, USA; 6 Contributing Editor, Alternative Medicine Review, Durango, CO, USA; 7 Institute for Agriculture and Trade Policy, Minneapolis, MN, USA; 8 Institute of Neurotoxicology and Neurological Disorders, 8232 14th Avenue NE, Seattle, WA, USA; 9 Shepherd University, Shepherdstown, WV, USA

## Abstract

Among dietary factors, learning and behavior are influenced not only by nutrients, but also by exposure to toxic food contaminants such as mercury that can disrupt metabolic processes and alter neuronal plasticity. Neurons lacking in plasticity are a factor in neurodevelopmental disorders such as autism and mental retardation. Essential nutrients help maintain normal neuronal plasticity. Nutritional deficiencies, including deficiencies in the long chain polyunsaturated fatty acids eicosapentaenoic acid and docosahexaenoic acid, the amino acid methionine, and the trace minerals zinc and selenium, have been shown to influence neuronal function and produce defects in neuronal plasticity, as well as impact behavior in children with attention deficit hyperactivity disorder. Nutritional deficiencies and mercury exposure have been shown to alter neuronal function and increase oxidative stress among children with autism. These dietary factors may be directly related to the development of behavior disorders and learning disabilities. Mercury, either individually or in concert with other factors, may be harmful if ingested in above average amounts or by sensitive individuals. High fructose corn syrup has been shown to contain trace amounts of mercury as a result of some manufacturing processes, and its consumption can also lead to zinc loss. Consumption of certain artificial food color additives has also been shown to lead to zinc deficiency. Dietary zinc is essential for maintaining the metabolic processes required for mercury elimination. Since high fructose corn syrup and artificial food color additives are common ingredients in many foodstuffs, their consumption should be considered in those individuals with nutritional deficits such as zinc deficiency or who are allergic or sensitive to the effects of mercury or unable to effectively metabolize and eliminate it from the body.

## Background

Neuronal plasticity, the ability of neurons to undergo specific functional changes during the moments when learning takes place, appears to underlie learning capacity [1]. Neuronal plasticity is crucial to the creation and storage of long-term memory. Dysfunction of neuronal plasticity is a factor in poor neural development [2-5]. Abnormal neuronal plasticity also has also been implicated in mental retardation, and autism [2-5]. Essential nutrients, including trace minerals, amino acids, and fatty acids, are necessary for proper functioning of the central nervous system and play a role in the maintenance of normal neuronal plasticity. Specifically, dietary deficiencies of iron, zinc, iodine, selenium, copper, manganese, fluoride, chromium, and molybdenum are associated with mild to significant changes in neuronal function that can lead to poor health and adverse effects on behavior and learning [6].

**Figure 1:**
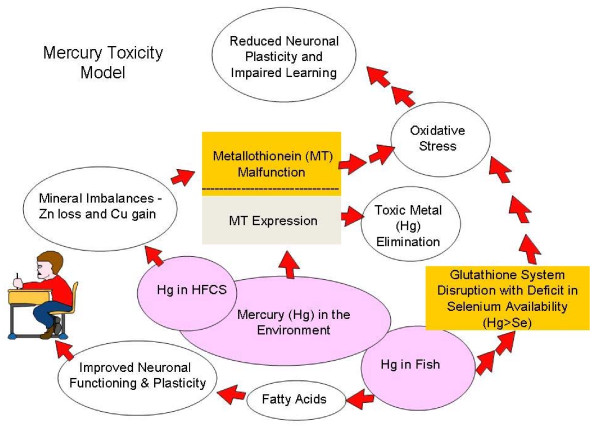
**Mercury Toxicity Model**.

Zinc deficiency related to dietary intake may be a factor in the development of several conditions that ultimately make learning more difficult for children. Such conditions include Autism Spectrum Disorders (ASD), Attention Deficit Hyperactivity Disorder (ADHD) and hyperactivity. In a recent review article, it was hypothesized that the increase in Autism may be related to transient maternal hypothyroxinemia resulting in low tri-iodothyronine (T3) in the fetal brain from insufficient dietary iodine intake and/or environmental exposure to antithyroid agents such as mercury [7]. Zinc deficiency, however, also lowers T3 [8] and may occur over time with the consumption of certain food chemicals such as high fructose corn syrup [9], tartrazine and sunset yellow [10,11]. The prevalence of low birth weight increased significantly in women in the United States (US) who had serum zinc levels in the lowest quartile [12], and low zinc intake during pregnancy is associated with a significant increase in the risk of preterm delivery [13]. Low birth weight and pre-term birth increase the risk of autism two fold [14]. Zinc is particularly crucial to a number of biological processes, and essential for neural development [15]. Poor nutrition leading to dietary deficiencies of zinc and/or other essential dietary nutrients such as selenium, amino acid methionine, and essential fatty acids can disrupt metabolic processes and impair brain function and neuronal plasticity by exacerbating heavy metal neurotoxicity [1,16-18]. There is evidence to suggest that the body's ability to maintain neuronal plasticity when essential dietary nutrients are lacking can be additionally impaired by exposure to environmental mercury. This article provides a review of such evidence and a model of how this toxic effect of mercury may occur (Figure 1).

The "Mercury Toxicity Model" in figure 1 is a flow chart of what can happen in the body when there is exposure to mercury. Neither the form of mercury nor the route of exposure is necessarily relevant for the purposes of this discussion. Exposure can occur via inhalation or ingestion; the model gives two examples of mercury (Hg) exposure from ingestion of foods found in our environment (Hg in Fish and Hg in High Fructose Corn Syrup), without excluding other possible exposures such as Hg in ambient air. Upon exposure, in a healthy individual with adequate nutrition, metallothionein is expressed and mercury is eliminated from the body. If the individual consumes an unhealthy diet leading to mineral imbalances, especially zinc loss and copper gain, then it is possible that either there will not be enough metallothionein to eliminate the mercury or the metallothionein may not function properly. In either case, if the mercury is not eliminated then it will lead to oxidative stress. An important target organ of mercury regardless of form when it cannot be eliminated from the body is the brain. In the case of ingestion, bacteria and yeast in the gut may change inorganic mercury to organic methyl mercury, which is thought to pass through the blood brain barrier. In brain tissue organic mercury may then be converted back to inorganic mercury. Oxidative stress in the brain from mercury insult leads to reduced neuronal plasticity and this impairs learning. Mercury exposure via fish consumption is a special case. Fish is an important dietary food as it contains the fatty acids important for improved neuronal functioning and plasticity. However, if fish does not contain selenium in a 1:1 ratio with mercury and there is more mercury in the fish than selenium, then it is possible that consumption of the fish can disrupt the glutathione system leading to oxidative stress caused by mercury insult or other neurotoxins. Disruption in the glutathione system also reduces neuronal plasticity and impairs learning. Properly functioning metallothionein and glutathione systems are therefore required for mercury metabolism. This model is not perfect but the following review provides evidence to support the hypothesis, that if correct, cannot be ignored for very long.

## Sources of mercury exposure

### Mercury in the environment

Mercury exposure, in its various forms, is thought to be a risk factor in causing some of the more prevalent neurological learning disorders, including autism [19]. A study of mercury in the environment and its correlation to pediatric neurodevelopmental disorders found that on average, for every 1,000 pounds of mercury (all forms) released into the environment via air emissions or wastewater effluent, as documented by the Environmental Protection Agency's Toxic Release Inventory, there was a 43-percent increase in the rate of special education services and a 61-percent increase in the rate of autism [20]. In a more recent study, investigators of environmental mercury exposure found that for every 1,000 pounds of mercury release, there is a 3.7% increase in autism rates of school age children living near coal fired power plants [21].

The presence of mercury in our environment will increase as long as humans continue to burn coal and use mercury to manufacture products (e.g., mercury amalgam, chlor-alkali chemicals, thermometers, thermostat switches). With respect to mercury emissions into the atmosphere, the United States Environmental Protection Agency (USEPA) estimates that about one third of US emissions are deposited within the contiguous US and the remainder enters the global cycle, of which the US contributes roughly three percent [22]. Current estimates are that less than half of all mercury deposition within the US comes from US emission sources. Mercury is therefore a transboundary issue and a global environmental contamination problem. In addition to the emissions from burning coal, mercury also enters the environment via wastewater from dental clinics using mercury amalgam and chlor-alkali chemical manufacturing plants using mercury cells. For environmental mercury levels to fall, there must be a worldwide effort to reduce mercury emissions from coal-fired power plants and to reduce the use of mercury in products and manufacturing processes.

With respect to mercury cell chlor-alkali chemicals, there are approximately 50 manufacturing plants left worldwide. In the US, many plants have closed and most of them are now abandoned Superfund sites or undergoing corrective clean up action for mercury contamination [23]. The mercury contamination is a result of many years of mercury loss to the building infrastructure, atmosphere, surrounding soil, groundwater, and nearby streams. Clean up of these sites is slow often taking many years as mercury is continually released into the environment [23-25] and clean up technology is still under development [23]. The exact amount of mercury released from these abandoned sites each day remains unknown. The mercury cell chlor-alkali manufacturing plants still in operation in the US use approximately 80 tons of mercury every year to manufacture the chlor-alkali products caustic soda, chlorine, potash, and hydrochloric acid [26]. These chlor-alkali products all contain residual mercury [27] and are used by food, chemical, and pharmaceutical manufacturers to make thousands of other products [28]. Although only five plants now operate in the US, mercury cell chlor-alkali chemicals continue to be imported by US manufacturers to make their products [29]. Because of the magnitude of the use of mercury in manufacturing products, mercury and mercury-containing compounds from these products are found as a waste material in both household and industrial wastewater effluent and in sewage sludge that can then be applied to land as a fertilizer [30].

As mercury is discharged into the environment by different industrial processes and through the use of mercury contaminated chlor-alkali chemicals, humans become exposed via the water they drink, the air they breathe, and the foods they eat. Chen et al. found that human subjects living in a mercury contaminated region had a mean mercury concentration in serum nearly 40 times that of the control group and a mean mercury concentration in urine almost 75 times that of the control subjects living in a non-contaminated region [31]. The mercury in the contaminated region came from different sources: mercury mining and ore processing, coal combustion for power production, and chlor-alkali industries [31]. Chen et al. determined that the human subjects living in the contaminated region were primarily exposed to mercury via the inhalation of elemental mercury vapor and the consumption of mercury contaminated foodstuffs, which contained different mercury species or forms [31].

With regard to cumulative mercury exposure, there are multiple possible sources. These sources may include mercury as a pollutant in air, soil, dust, and water, a contaminant in foodstuffs and consumer products, a material in dental amalgam and lighting fixtures, and a contaminant in fish and seafood. Overall environmental mercury exposure includes all of these sources as "environment" includes home and office as well as the outdoors. Given the growing evidence of ongoing, cumulative environmental mercury exposure, it may be prudent to follow the American Academy of Pediatrics' guidelines for limiting exposure to all forms of mercury to help prevent neurodevelopmental problems in children [32].

### Methyl mercury in fish: a special consideration

Fish is an important dietary source of omega-3 fatty acids that are required for normal neural development. However, fish can also be contaminated with mercury. (Figure 1.) According to the World Health Organization (WHO) and the Food and Drug Administration (FDA), concentrations of methylmercury (MeHg) in various species of fish cover a wide range, from less than 0.01 ppm to over 3.0 ppm fresh weight, depending on factors such as pH, the redox potential of the water, and the species, age and size of the fish [33,34]. Human hair analysis for mercury is a useful biomarker for determining long-term exposure to mercury from fish and non-fish food. Using data from the 1999-2000 National Health and Nutrition Examination Survey (NHANES), McDowell et al [35] found that total hair mercury is associated with age, race/ethnicity, and frequency of fish consumption. Analyses of blood from participants of the NHANES also found elevated mercury levels in women who were older, reported eating more fish, and who designated themselves in the "other" racial/ethnic category (includes Asians, Native Americans, and Pacific Islanders [36,37]. With respect to chronic, low-level exposure to MeHg from maternal seafood consumption during pregnancy, there have been two studies published with conflicting results. In the Faroe Islands study, researchers found after following 900 children until seven years of age that higher umbilical cord blood MeHg was associated with lower scores on several developmental and cognitive tests [38]. This finding differs from the Seychelles Child Development study that found no association between total maternal hair mercury and the neurodevelopmental test performance demonstrated by 700 children, who were followed until up to nine years [39]. These differing findings have led to much controversy as to whether the risks of low-level MeHg exposure outweigh the benefits provided by omega-3 fatty acids in fish. The controversy, however, is based on the assumption that dietary mercury exposures were the same for mothers who participated in the Seychelle and Faroe Island studies. Dietary mercury exposures for these two groups are not the same however. The Faroese diet includes whale meat and the Seychelles Islanders' diet does not [40]. In whale meat, the concentration of mercury rises continually with age and can exceed the selenium content [41]. Selenium is an important micronutrient needed to support glutathione function, which protects neurons from damage caused by mercury induced oxidative stress (figure 1). In fish, the concentration of mercury also rises continually with age but does not generally exceed the selenium content [42]. The Faroese diet of whale meat most likely contains more unmitigated mercury exposure with lower levels of selenium.

Studies suggest there is an interaction between mercury and selenium [43-46], however, it is unclear whether selenium protects from the toxic effects of mercury or mercury interferes with the benefits of selenium. It has been suggested that the formation of a 1:1 Hg-Se compound may explain the mercury detoxification by selenium [46]. In the study referenced earlier, Chen et al. reported serum selenium concentrations were significantly higher in the human subjects living in the mercury-contaminated region [31]. Specifically, with increased exposure to all forms of environmental mercury, serum selenium concentrations associated with glutathione peroxidase (GSH-Px) were 2 times higher in the mercury-exposed group than in the control group living in the non-contaminated area [31]. Further research is needed to determine how selenium and mercury interact when it comes to environmental mercury exposure or their concomitant consumption in fish or whale meat.

The results of a 2005 study found that, with moderate fish consumption during pregnancy, there was a benefit to offspring cognition, but that exposure to higher levels of mercury from fish led to adverse effects on child cognition [47] (Figure 1). The consensus now is that pregnant women do not need to avoid eating fish, but they need to choose varieties of fish with lower mercury concentrations [48]. Unfortunately, many species of fish have been compromised by MeHg contamination and the USEPA and the FDA have issued a joint advisory to pregnant women and others on the hazards of overconsuming contaminated commercial and non-commercial fish [49]. The most contaminated fish include king mackerel, swordfish, shark and tilefish.

Fish advisories for mercury in the US increased from 2,436 in 2004 to 3,080 in 2006. Thirty-five of fifty states have issued statewide fish advisories for methylmercury that suggest limiting consumption of certain fish and some include recommendations for safe levels of consumption and/or exposure [50]. These advisories often focus on the health effects of mercury exposure associated with neurodevelopmental harm and are therefore limited to the infant and women who are pregnant, nursing, or of reproductive age. No studies have been conducted to determine what effect fish consumption may have on sensitive populations such as children with attention deficit hyperactivity disorder (ADHD) or some form of autism spectrum disorder (ASD), however, clinical trials have been performed giving children with ADHD fish oil as a fatty acid supplement. The results of a study by Richardson and Puri are discussed in the last section of this article [51].

### Total mercury in high fructose corn syrup

A key discovery related to the isomerization of dextrose in the 1960's led to the development of high fructose corn syrup (HFCS) by the corn refining industry [52]. HFCS is the end product from a corn wet-milling process that involves a number of steps in a product line that yields corn oil, animal feed, starch products, and corn sweeteners. Food manufacturers use HFCS as a sweetener to stabilize food products and enhance product shelf life [53]. Several chemicals are required to make HFCS, including caustic soda, hydrochloric acid, alpha-amylase, glucoamylase, isomerase, filter aid, powdered carbon, calcium chloride, and magnesium sulfate [54]. Up until recently, HFCS manufacturers used "mercury grade" caustic soda from mercury cell chlor-alkali plants to make their product and as a result consumers were likely exposed to low levels of mercury over time [55]. Using the results of analytical testing conducted on FDA collected samples of commercial HFCS for total mercury and the US per capita consumption of HFCS, Dufault et al. estimated daily mercury intakes from HFCS ranging up to 28 ug [55,56]. Consumption of mercury contaminated HFCS may have over time exceeded other major sources of mercury especially in high-end consumers of beverages sweetened with HFCS [55]. In response to the Dufault et al finding, the Corn Refiners Association stated in a news interview that the findings were outdated because they no longer use mercury cell chlor-alkali products in their manufacturing process [57]. This change in manufacturing practices has not been verified by a third party with nothing to gain outside of the corn industry and there are no regulations in place to prevent the future use of mercury cell chlor-alkali products in the HFCS manufacturing process. In any case, the data released by Dufault et al remain the only publicly available peer reviewed data. The Corn Refiners Association does not appear to dispute the fact that for many years, HFCS consumers were exposed to low levels of mercury in their diet from this product. HFCS is now ubiquitous in processed foods and significantly consumed by people all over the world. By the mid 1980's, when high fructose corn syrup had become the sweetener of choice by the soft drink beverage industry [52] as well as the manufacturers of many other processed food products, there was a corresponding rise in the prevalence of autism [58].

The rates of diagnosed Autism Spectrum Disorders are shown in Table 1 for California, the only State that reports number of cases dating back to the mid-1980s [59]. The annual net growth in cases ranges from 9% to 12% between 1987 and 2006-2007 and is consistent with growth found in other states in recent years. Annual growth rates of Autism Spectrum Disorder in California peaked in 2001 and 2002 at 19.6 and 19.4 percent respectively then declined to 11.8 percent in 2006. Once a child is diagnosed with autism, the diagnosis is stable as is shown by the data from a longitudinal study by Lord and her colleagues [60]. In that study fewer than 6% of the children ages 2 to 9 had a reversal in the diagnosis. Similarly, in California, 94 percent of total persons with Autism Spectrum Disorders kept their diagnoses. Table 1 also shows the per capita consumption of high fructose corn syrup in pounds per year based on USDA estimates. The peak years for annual consumption of HFCS occurred in 2000-2002 at 44.6 and 44.8 lbs/yr respectively, the same years as the peak for autism.

**Table 1:** Net Growth in Persons Diagnosed with Autistic Spectrum Disorders, CA Per Capita Consumption of High Fructose Corn Syrup, US, 1987-2006

**Year**	**% Growth** **Autism**	**Per Capita** **Consumption** **HFCS, lb/yr**
1987	9.0	34.0

1988	10.8	34.9

1989	11.3	34.3

1990	10.9	35.3

1991	10.4	35.8

1992	8.5	36.9

1993	12.9	38.8

1994	13.5	40.0

1995	13.4	41.0

1996	16.8	41.1

1997	17.2	43.0

1998	17.2	44.1

1999	16.2	45.4

2000	18.3	44.6

2001	19.6	44.6

2002	19.4	44.8

2003	13.5	43.4

2004	12.1	42.7

2005	10.6	42.2

2006	11.8	41.5

CA = California; US = United States; % = percent; HFCS = High Fructose Corn Syrup; lb/yr = pounds per year

The decline in annual growth rates of Autism Spectrum Disorder in California could be explained by the reduction in annual per capita consumption of high fructose corn syrup or the reported reduction in the use of mercury cell chlor-alkali chemicals in the high fructose corn syrup manufacturing process. Whatever the case, there may be a biochemical connection between low level mercury exposures, zinc deficiency and the development of learning disorders such as ADHD and autism spectrum disorders. In a 1990 human study, researchers found that consumption of fructose and HFCS may lead to certain mineral imbalances including zinc loss and copper gain [9] (Figure 1). The human subjects demonstrated a reduction in numerical zinc balance and an increase in numerical copper balance when they were fed fructose and HFCS compared to the adjustment period [9]. This finding along with the more recent finding of the historic use of mercury in high fructose corn syrup [55] is of particular significance today with respect to neurodevelopmental disorders since there is now evidence that autism can be caused by a biochemical abnormality that disables the metal clearing function of the zinc dependent metallothionein (MT) protein [61-64] and zinc deficiency plays a role in ADHD [10,11]. Two mechanisms with the potential for disabling MT functioning include severe zinc depletion [65] and toxic metal overload [61-64]. Long term exposure to mercury contaminated high fructose corn syrup could produce both of these mechanisms and/or conditions that may disable MT functioning and lead to the bioaccumulation of mercury or other adverse neurological effect.

Children with Autism Spectrum Disorders have increased body burdens of mercury [66]. Because mercury inhibits cysteine ligands in the metal clearing metallothionein (MT) proteins that normally bind with metal ions such as copper and zinc [67], consumption of mercury-contaminated HFCS and other zinc depleting substances [10,11] over time by sensitive individuals may induce an MT malfunction (Figure 1). Such a malfunction may lead to severe zinc deficiency and allow copper to reach toxic levels in membranes leading to lipid peroxidation and cell damage [68] (Figure 1). Exposure of the brain to excessive oxidative stress may lead to a loss in learning capacity (Figure 1). Many children with ADHD are deficient in the micronutrient zinc [69,70]. Consumption of zinc depleting food additives [9-11] increases hyperactive behaviors (inattention, impulsivity, and over activity) both in children with extreme hyperactivity (i.e., ADHD) and in the general population [71]. Increased hyperactivity is associated with the development of educational difficulties especially in reading [72]. In rats, zinc deficiency along with oxidative stress predisposes the brain to damage by disruption of the blood-brain barrier [73] and prenatal zinc deficiency has pronounced effects on postnatal metallothionein metabolism, which can persist into adulthood [65]. With respect to consumption of high fructose corn syrup and adverse effect, Molteni et al. [74], found that a high fat, HFCS diet reduced brain-derived neurotrophic factor (BDNF) neuronal plasticity, and learning in rats. BDNF levels have been measured in human subjects with neurodevelopmental learning disorders. In 2006, Hashimoto et al. reported the serum levels of BDNF in human patients with autism were significantly lower than those of normal controls [75]. Shim et al. however, found mean plasma BDNF levels were significantly higher in 41 ADHD patients compared to 107 normal controls and suggested that plasma BDNF levels were positively associated with the severity of inattention symptoms [76]. Further studies will be required to understand the role of BDNF in either learning disorder.

As indicated by our toxicity model (figure 1), mercury may adversely affect learning if either metallothionein and glutathione metabolic functions are not functioning properly and supported by key dietary nutrients. Both of these metabolic functions are required by the body's immune system to combat mercury exposure. How the immune system responds to mercury insult is discussed in the section below.

## Nutritional deficiencies and metabolic disruptions in sensitive populations

### Amino acids and the glutathione system

Essential amino acids, fundamental building blocks of proteins that provide the structural integrity of all living organisms, are provided by a diet rich in protein. Essential amino acids (those not produced endogenously) include histidine, isoleucine, leucine, lysine, methionine, phenylalanine, threonine, tryptophan, and valine. Essential amino acid deficiency can lead to adverse health effects. For instance, a deficiency of the essential amino acid methionine can adversely affect behavior and learning. Researchers found that 51 percent of autistic children showed evidence of methionine deficiency [77]. In a more recent study of 90 autistic children and 45 controls, mean plasma levels of methionine, cysteine, total glutathione, and the ratio of oxidized to reduced glutathione were significantly decreased among the autistic children [78]. Methionine acts as a precursor in the production of glutathione (GSH) in mammalian hepatocytes as a result of its conversion to cysteine [79]. James et al. found lower concentrations of methionine in the plasma of 20 autistic children compared to 33 control children without autism in a study published in 2004 [80].

GSH is the primary defense used by the neuron against free radicals. Depletion of GSH in rats can impair short-term and long-term mechanisms of synaptic plasticity and stress the redox balance in the normal function of brain circuitry [81]. Metals, particularly copper and mercury, play a primary role in the initiation of reactive oxygen species and the depletion of GSH [31,82].

**Figure 2:**
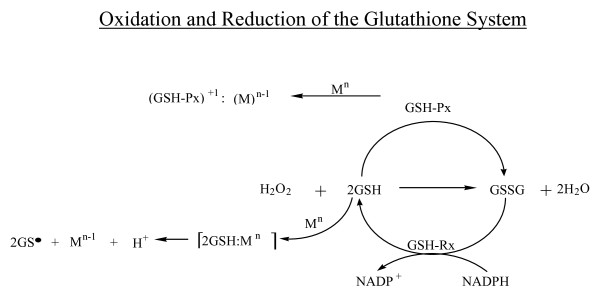
**Oxidation and Reduction of the Glutathione System**.

The GSH system is effective at scavenging free radicals and serves as an oxidation reduction buffer that allows for the reduction of the highly reactive hydrogen peroxide (H_2_O_2_; a byproduct of cellular respiration) to water. This prevents oxidative stress, which could lead to lipid peroxidation and cell damage [83,84]. In the reaction scheme illustrated in Figure 2, oxidation is potentiated by the catalyst glutathione peroxidase (GSH-Px) in which selenium plays a key role as an antioxidant cofactor. GSH-Px catalyzes the oxidation of reduced glutathione and allows for the reduction of hydrogen peroxide to water, preventing lipid peroxidation and cell damage [31,83,85]. Oxidized glutathione (GSSG) is reduced to GSH, a reaction catalyzed by glutathione reductase (GSH-Rx). Mercury or copper, shown as M^n ^in Figure 2, can disrupt the GSH system, leading to cellular insult and potential apoptosis as a result of the generation of hydrogen peroxide [86]. Results from the recent study by Chen et al. of mercury-exposed humans suggest that mercury affects the bioavailability and retention of selenium and interferes with the metabolic processes dependent on selenium [31]. The researchers found that the serum selenium concentrations associated with GSH-Px and selenoproteins were 2 times higher in the mercury-exposed group than the control group [31]. A disruption of the GSH system by mercury leads to GSH depletion and cell destruction. An *in vitro *study of Jurkat T cells exposed to thiomersal demonstrated concentration-dependent apoptosis [87]. It was found that the mercury moiety, not the thiosalicylic acid moiety, of thiomersal was responsible for glutathione depletion [87]. GSH depletion is linked to several neurodegenerative disorders [88]. James et al. found lower concentrations of total GSH in the plasma of 20 autistic children compared to 33 control children without autism in a study published in 2004 [80]. In a recent study published in 2009, Sitta et al. found erythrocyte and plasma glutathione levels were significantly reduced in phenylketonuric patients compared to a control group [89]. When the GSH system is upset by the presence of mercury, changes in the immune system occur. Mercury exposure activates the glucocorticoid anti-inflammatory response [90,91]. Glucocorticoids enhance the neurotoxicity of reactive oxygen species by decreasing the activity of glutathione peroxidase. This results in a negative feedback loop that depletes GSH and exacerbates the apoptotic process [92,93] (Figure 2). The glucocorticoid anti-inflammatory response also induces MT expression in the presence of mercury [91,93-96].

### Metallothionein: a protein for metal elimination

MTs represent a family of ubiquitous, low-molecular-weight, metal-binding proteins that have a molecular mass of less than 7,000 Daltons, containing approximately 33-percent cysteine residues [94]. Due to their binding capacity (i.e., up to seven zinc or 12 copper ions per molecule) and ubiquitous nature, it has been suggested that MTs may engage in essential metal trafficking to and from many metal-dependent proteins [91]. MTs are involved in many important processes such as the regulation of zinc homeostasis, necessary for proper enzyme function [95-97]. Although as many as 18 different metals may be associated with an MT molecule, only copper, cadmium, lead, silver, mercury, and bismuth are capable of displacing a zinc atom [94]. All of these known environmental toxicants cause MT expression [94]. MTs are among the body's primary protective molecules against toxic metals [68,98]. In mice, MT provides a survival benefit in stressful situations, such as heavy metal and free radical exposure, inflammation, and zinc or other nutrient deficiency [94]. The protective effect of MT against the toxicity of cadmium and mercury vapor has been the most studied thus far [99-102].

Besides toxic metal exposure, the MT gene family is also induced by bacterial cell wall lipopolysaccharides and inflammatory cytokines (such as interleukin-1beta), collectively playing a major neuroprotective role against these various environmental insults. Constitutive cellular abundance of MTs -1, -2, -3 and -4 (the latter specifically localized to stratified squamous epithelium) are therefore an important index of a tissue's susceptibility, and capability to handle a wide array of toxic insults. As the nasal cavity, primary olfactory neurons at the cribiform plate, and anatomically connected olfactory bulb are continually exposed to various airborne pollutants in the external environment, the olfactory system provides a direct route of entry into the central nervous system for an assortment of environmental neurotoxic agents including mercury. It is therefore not surprising that the olfactory system has one of the highest MT abundances of the mammalian central nervous system, and that expression of the MT-1, -2, and especially -3 isoforms are greatest in the olfactory neurons of the brain and the anatomically adjacent hippocampal region, which has direct nerve fiber projection contacts from the olfactory bulb [94,101,103-105]. In mercury vapor intoxicated mice, MT-3 expression was found to dominate in the olfactory cells of the olfactory mucosa as well as in neurons of the olfactory bulb, while MT-1 and -2 immunoreactivity predominated in supporting basal and acinar cells of the Bowman's gland of the olfactory mucosa [101].

MT-3 is a brain specific, high zinc containing MT, which is found in neuronal and glial cells [94,101,104-107]. Researchers examined 503 patients with autistic spectrum disorders and found lower levels of MT with significantly higher copper: zinc ratios in the autistic group compared to healthy age- and gender-matched controls [77]. Three peer reviewed papers published in 2009 report findings that support the link between low-dose mercury exposure and metallothionein dysfunction and an association with ASD [62-64]. It has been theorized that MTs may serve as a redox control and the fact that MT-3 is relatively high in zinc and is associated with neurons provides evidence that it fills a role as an oxidation-reduction buffer in neuronal tissue. Importantly, the MT protein in the immune system may not function properly if either zinc or methionine are deficient in the diet [108,109]. Consumption of any substance leading to zinc loss and copper gain (e.g. HFCS) or zinc deficiency (e.g. tartrazine and sunset yellow food dye) should be avoided by sensitive populations such as children diagnosed with ASD, ADHD or found to be zinc deficient.

### Fatty acids and neuronal functioning

Omega-3 fatty acids have been found to be a basic component of normal neural development and maintenance of neural plasticity [110]. Dietary sources of omega-3, however, are not plentiful in plant foods with the exceptions of flax (*Linum usitatissimum) *and Chia (*Salvia hispanica*) seeds, and are found in small amounts in walnuts, soybeans, canola oil, and free-range chicken eggs [110]. The only rich sources of omega-3 fatty acids are cold water fish (e.g., anchovies, krill, herring, tuna, mackerel, salmon, and sardines), cod liver oil, and other fish oils. Deficiencies of two important omega-3 fatty acids, eicosapentaenoic acid (EPA) and docosahexaenoic acid (DHA), commonly lead to adverse health effects and have been found in children with behavior disturbances [111,112].

The role of DHA in brain development and visual acuity has been extensively studied [113]. The breast milk of nursing mothers contains adequate amounts of DHA if dietary intake is sufficient [113]. There is a preponderance of evidence that shows infants fed unfortified formula have poorer vision and lower IQs than infants fed formula fortified with DHA [113]. In 1993, the WHO recommended that pre-term and term infant formulas include DHA and in 2001, FDA allowed a manufacturer's self-declaration that DHA and arachidonic acid (AA) were generally recognized as safe (GRAS) for use in infant formula [114]. In 2002, infant formula manufacturers began marketing infant formulas containing DHA and AA in the US.

DHA protects neurons and glia from death, in part, by maintaining BDNF, which is a small protein, made within the brain that is crucial for maintaining neuronal plasticity [115,116]. Wu et al. [117] found that dietary omega-3 fatty acids in the form of DHA not only normalize BDNF but also reduce oxidative damage and counteract learning disability after traumatic brain injury in rats. Oxygenated DHA-derivatives, including a recently described form of modified DHA called neuroprotectin D1, have been shown to promote brain cell survival via the induction of highly specific brain gene expression programs that confer strong resistance to apoptosis and potent neuroprotection [118].

Researchers have been investigating the link between essential fatty acids and ADHD in children. Stevens et al. [119] found that 53 children with ADHD had significantly lower plasma levels of omega-3 fatty acids than did the control group of 43 children. The authors noted that the children with the worst symptoms of essential fatty acid deficiency increased thirst, frequent urination, dry hair and skin - had the lowest plasma levels of omega-3 fatty acid levels. Another study, conducted by Mitchell et al. [120], compared 44 hyperactive children with 45 age- and sex-matched controls. DHA levels were significantly lower in the hyperactive children, who also had auditory, visual, language, reading, and learning difficulties, and lower average birth weight compared to controls. Colter et al. [121] also found low levels of DHA and total omega-3 fatty acids in ADHD children compared with controls. In an effort to raise DHA levels, Voight et al. [122] administered 345 mg per day of DHA to 27 children with ADHD without any significant improvement in behavior compared to the 27 children who served as controls. Hirayama et al. [123] used a higher dose of DHA (514 mg per day) than the previous study and also found no improvement in the DHA group.

Despite the fact that low levels of DHA were found in children with ADHD, supplementing with DHA alone or in high amounts relative to other essential fatty acids (EFAs) have not been shown to be effective in improving behavior. Researchers have begun to look at other fatty acids and found that a combination of EFAs and in particular EPA may be more effective in the modulation of behavior disorders. Forty-one children with learning difficulties were randomly assigned to supplementation with EPA 186 mg, DHA 480 mg, gamma linolenic acid (GLA) 96 mg, linoleic acid 864 mg, and AA 42 mg or placebo (olive oil) for 12 weeks [51]. The group receiving the fish oil supplement had significant improvements in learning and behavioral scores and other ADHD-related symptoms compared to the group given olive oil [51]. (Figure 1.) In a later study, 117 children with developmental coordination disorder were given either 558 mg EPA, 174 mg DHA, and 60 mg GLA or placebo [124]. The children receiving the fish oil supplement demonstrated significant improvements in reading, spelling, and behavior after three months of treatment compared to placebo [124]. In an open-label pilot study, Sorgi et al. [125] gave a high dose EPA/DHA supplement containing 10.8 g of EPA and 5.4 g of DHA per day to 9 children for eight weeks. They found significant improvements in behavior including inattention, hyperactivity, oppositional behavior and conduct disorder. Sinn and Bryan [126] administered 558 mg EPA, 174 mg DHA and 60 mg GLA/day to 36 children for 15 weeks and improvements were found for inattention and hyperactivity and impulsivity compared with the control group. Recently both Johnson et al. [127] and Belanger et al. [128] found improvement in inattention in a subgroup of children with ADHD when supplemented with a mixture of these essential fatty acids.

EPA and DHA are both essential for optimal brain function, but for different reasons. DHA is important for the structure of the neuronal membranes, while EPA plays a role in brain function by regulating neuronal signaling, neurotransmitter uptake, and the activity of phospholipase enzymes. EPA has been shown to reduce the overactivity of phospholipase A_2 _which in excess can deplete the phospholipids of the long chain polyunsaturated fatty acids critical to normal brain function. EPA may be a useful adjunct in the treatment of various neurological conditions including depression, bipolar disorder, schizophrenia, dyslexia, dyspraxia, ADHD, and Autism Spectrum Disorders [112,129,130].

Magnesium and zinc are needed to help convert the 18-carbon, plant-derived essential fatty acids (EFAs) to long chain fatty acids, notably DHA and EPA [131]. Many children with ADHD are deficient in these nutrients and may therefore have difficulty elongating the 18-carbon fatty acids [132]. In a sample of 116 children with ADHD, researchers found that 95 percent had a magnesium deficiency [133]. In 1997, researchers found that a combination of magnesium aspartate and magnesium lactate supplemented at a dose of 200 mg daily for six months significantly reduced disruptive behavior in children with ADHD compared to a control group [134]. Galland [135] found an impaired desaturation of polyunsaturated fatty acid in a magnesium compromised population. This desaturation enzyme is magnesium dependent. Mahfouz et al. [136] found a significant reduction in long chain polyunsaturated fatty acids in magnesium deficient cells pointing to an impairment of delta 5 and/or delta 6 desaturase enzymes. It is possible that correcting a magnesium deficiency may restore desaturase enzyme function. Researchers also found that deficient levels of zinc are more commonly found in children with ADHD and supplementing these children with zinc significantly reduced hyperactivity, impulsivity, and impaired socialization scores, especially in children with low levels of EFAs [137,138].

Children who have difficulty converting EFAs to long chain fatty acids can obtain a preformed source of EPA and DHA as a nutritional supplement or in the diet by consuming fish or fish oil. As discussed above, cold-water fish provide the only rich dietary source of these omega-3 fatty acids; however, recent research on trace metal contamination has questioned their safety. Guallar et al. [139] found, for instance, that the cardio protective benefits of fatty fish may be compromised by their high mercury content. Canadian researchers found a significant association between high levels of mercury and EPA in the blood of children (consistent with high levels of fish consumption) [140]. In addition, the researchers found a statistically significant inverse association between attentional focusing and blood mercury levels in children under age three years of age [140]. A careful selection of fish with low mercury and high EFA content, such as salmon and sardines, is essential in minimizing exposure to mercury while increasing consumption of these important EFAs. With respect to the consumption of fish oil or other supplements that are fish-derived, caution is warranted, as mercury contamination may be possible. Out of 100 samples of fish oil dietary supplements analyzed for mercury by the Food Standards Agency in the United Kingdom, nine were found to contain mercury above the limit of detection of 0.0014 mg/kg [141].

## Key issues related to mercury exposure guidelines

While the total dietary intake of mercury is not known, mercury is widely accepted to be an unusually toxic heavy metal and the American Academy of Pediatrics has recommended that minimizing exposure to any form of mercury is essential for optimal child health [32]. The current recommendation for "safe" mercury consumption is based on a "reference dose" or RfD from fish consumption and only applies to methyl mercury (MeHg). The USEPA and FDA define the RfD as " [a]n estimate (with uncertainty spanning perhaps an order of magnitude) of a daily oral exposure to the human population (including sensitive subgroups) that is likely to be without an appreciable risk of deleterious effects during a lifetime." The current official RfD for methyl mercury in the US is set at 1 ppm although EPA has historically pushed for a lower reference dose of 0.1 ppm. Unfortunately, this official "reference dose" of 1 ppm does not take into consideration what the human body needs nutritionally to successfully metabolize and excrete methyl mercury, and it only applies to MeHg exposure.

With regard to total mercury exposure, the Joint Expert Committee on Food Additives (JECFA) recommends an exposure limit of 1 ppm for mercury in their 2005 Evaluation of Food Additives report [142]. This report does not specifically address the mercury content in the food color additives tartrazine also known as FD&C Yellow 5 or sunset yellow also known as FD&C Yellow 6 but these chemicals are manufactured with the chlor-alkali product "hydrogen chloride" and may not contain more than 1 ppm mercury according to US Food and Drug Administration regulations [143]. In addition to causing zinc deficiency, both of these food color additives have been linked to hyperactivity in children [71] and the United Kingdom has asked manufacturers to voluntarily ban their use in food products [144].

## Etiology of ASD and ADHD

In 2007 alone, there were 1000 studies published on all aspects of ASD [145]. As is the case with ADHD, there are likely unknown and unmeasured variables associated with the development of ASD. Disease related and/or exposure related bias arises when there is a causal or associational path of no interest to the researchers [146]. In this review article we have clearly not identified all of the possible variables associated with the development of these two disease conditions. The development of ASD and ADHD likely involve a broad range of genetic, prenatal, social, developmental, nutritional and environmental factors [145,147]. We have focused primarily on some of the nutritional factors that may be considered along with environmental mercury exposure in the development of treatment plans for patients with ASD or ADHD. Multiple treatment modalities are most likely needed to treat these patients successfully [147].

## Conclusion

Adequate nutrition, specifically omega-3 fatty acids, methionine, and the trace minerals zinc and selenium are important for maintaining neuronal plasticity and learning capacity. The evidence does suggest that improper or inadequate nutrients may lead to behavior and learning disorders when mercury is present in the environment. Because current standards allow the use of mercury cell chlor-alkali chemicals in food manufacturing, food products that are made with one or more of these ingredients may contain trace amounts of mercury such as is the case for HFCS and other food additives. These low-level mercury exposures may contribute to cumulative environmental mercury exposure and present a problem for sensitive individuals who do not have adequate levels of glutathione and metallothionein and/or are deficient in the trace minerals needed for metal metabolism. Because consumption of HFCS and other food additives may lead to trace mineral imbalances and/or zinc deficiency, further research is needed to determine whether or not these food ingredients are safe for consumption by sensitive populations. If food manufacturers continue to use mercury grade chlor-alkali chemicals, it may be necessary to account for this source of mercury exposure in dietary guidelines for sensitive populations such as children with ADHD and autism spectrum disorders. In addition, consideration of dietary micronutrients required to enhance neurological development and support proper functioning of metallothionein, glutathione peroxidase, and other metal clearing and detoxifying mechanisms in the brain seems warranted.

## List of abbreviations

ASD: Autism Spectrum Disorder; ADHD: attention deficit hyperactivity disorder; T3: tri-iodothyronine; US: United States; USEPA: United States Environmental Protection Agency; WHO: World Health Organization; FDA: Food and Drug Administration; MeHg: methylmercury; NHANES: National Health and Nutrition Examination Survey; GSH-Px: glutathione peroxidase; HFCS: high fructose corn syrup; ug: micrograms; MT: metallothionein; BDNF: brain-derived neurotrophic factor; GSH: glutathione; GSSG: oxidized glutathione; GSH-Rx: glutathione reductase; EPA: eicosapentaenoic acid; DHA: docosahexaenoic acid; AA: arachidonic acid; GRAS: generally recognized as safe; GLA: gamma linolenic acid; EFAs: essential fatty acids; mg/kg: milligrams/kilogram; RfD: reference dose; ppm: parts per million; JECFA: Joint Expert Committee on Food Additives.

## Competing interests

The authors declare that they have no competing interests.

## Authors' contributions

RD spearheaded the review and recruited interdisciplinary collaborators to contribute to the development of the manuscript. RD was the lead environmental investigator and literature reviewer for the sources of mercury exposure section. LP and BL both contributed greatly to the development of the amino acids and glutathione system section. WL was the lead investigator and reviewer for the metallothionein section. RS was the lead investigator and reviewer for the fatty acids section. RC reviewed, analyzed, and prepared the epidemiological data. CC, DW, and SG were instrumental in editing the manuscript and reviewing it critically for intellectual content. All authors read and approved the final manuscript.
