# Correction to: Mercury exposure, nutritional deficiencies and metabolic disruptions may affect learning in children

**DOI:** 10.1186/s12993-018-0136-9

**Published:** 2018-02-07

**Authors:** Renee Dufault, Roseanne Schnoll, Walter J. Lukiw, Blaise LeBlanc, Charles Cornett, Lyn Patrick, David Wallinga, Steven G. Gilbert, Raquel Crider

**Affiliations:** 1 United Tribes Technical College, Bismarck, ND USA; 2 Department of Health and Nutrition Sciences, Brooklyn College of CUNY, Brooklyn, NY USA; 3 Departments of Neuroscience and Ophthalmology, LSU Neuroscience Center, Louisiana State University Health Sciences Center, New Orleans, LA USA; 4 Carl Hayden Bee Research Center, Tucson, AZ USA; 5 Department of Chemistry and Engineering Physics, University of Wisconsin-Platteville, Platteville, WI USA; 6 Contributing Editor, Alternative Medicine Review, Durango, CO USA; 7 Institute for Agriculture and Trade Policy, Minneapolis, MN USA; 8 Institute of Neurotoxicology and Neurological Disorders, 8232 14th Avenue NE, Seattle, WA USA; 9 Shepherd University, Shepherdstown, WV USA

## Correction to: Behav Brain Funct (2009) 5:44 10.1186/1744-9081-5-44

The original version of this article [1] unfortunately contained an error which has since been acknowledged in this Correction article. The URL link in the Reference 19 was broken and it needs to be replaced with the active link given below.

The corrected Reference 19 is given below.

19. Institute for Children’s Environmental Health. https://www.blacknote.com/wp-content/uploads/2017/12/LDDIStatement-2.pdf.
